# Alcohol-associated liver disease: A review on its pathophysiology, diagnosis and drug therapy

**DOI:** 10.1016/j.toxrep.2021.02.010

**Published:** 2021-02-19

**Authors:** Vetriselvan Subramaniyan, Srikumar Chakravarthi, Ravindran Jegasothy, Wu Yuan Seng, Neeraj Kumar Fuloria, Shivkanya Fuloria, Iswar Hazarika, Anju Das

**Affiliations:** a Department of Pharmacology, Faculty of Medicine, Bioscience and Nursing, MAHSA University, SP 2, Bandar Saujana Putra, 42610, Malaysia; b Department of Pathology, Faculty of Medicine, Bioscience and Nursing, MAHSA University, SP 2, Bandar Saujana Putra, 42610, Malaysia; c Department of Obstetrics and Gynecology, Faculty of Medicine, Bioscience and Nursing, MAHSA University, SP 2, Bandar Saujana Putra, 42610, Malaysia; d Department of Biochemistry, Faculty of Medicine, Bioscience and Nursing, MAHSA University, SP 2, Bandar Saujana Putra, 42610, Malaysia; e Department of Pharmaceutical Chemistry, Faculty of Pharmacy AIMST University, Jalan Bedong-Semeling, 08100, Malaysia; f Department of Pharmacology, Girijananda Chowdhury Institute of Pharmaceutical Sciences, Guwahati, 781017, India; g Department of Pharmacology, Royal School of Pharmacy, Royal Global University, Guwahati, 781035, India

**Keywords:** ALD, alcohol associated liver disease, ALT, alanine aminotransferase, AST, aspartate aminotransferase, CD14, cluster of differentiation14, CHD, congenital heart disease, ECM, extracellualr matrix, FASD, fetal alcohol spectrum disorders, FDA, food and drug administration, GGTP, gamma-glutamyl transpeptidase, GSH, Glutathione, H_2_O_2_, hydrogen peroxide, HCV, chronic hepatitis C, HSC, hepatic stellate cells, IGR, intrauterine growth retardation, IL, interleukin, JECH, Japan Environment and Children's Study, MDF, maddrey’s discriminant function, NA, nutritional assessment, NAC, N-acetylcysteine, NADPH, Nicotinamide adenine dinucleotide phosphate, OLT, Orthotopic liver transplantation, ROS, reactive oxygen species, TLR4, toll-like receptor 4, TNF, Tumor necrosis factor, Alcohol, Liver pathogenesis, Pregnancy, Immune modulation, Targeted therapy

## Abstract

One of the global burdens of health care is an alcohol-associated liver disease (ALD) and liver-related death which is caused due to acute or chronic consumption of alcohol. Chronic consumption of alcohol damage the normal defense mechanism of the liver and likely to disturb the gut barrier system, mucosal immune cells, which leads to decreased nutrient absorption. Therapy of ALD depends upon the spectrum of liver injury that causes fatty liver, hepatitis, and cirrhosis. The foundation of therapy starts with abstinence from alcohol. Corticosteroids are used for the treatment of ALD but due to poor acceptance, continuing mortality, and identification of tumor necrosis factor-alpha as an integral component in pathogenesis, recent studies focus on pentoxifylline and, antitumor necrosis factor antibody to neutralize cytokines in the therapy of severe alcoholic hepatitis. Antioxidants also play a significant role in the treatment but till today there is no universally accepted therapy available for any stage of ALD. The treatment aspects need to restore the gut functions and require nutrient-based treatments to regulate the functions of the gut system and prevent liver injury. The vital action of saturated fatty acids greatly controls the gut barrier. Overall, this review mainly focuses on the mechanism of alcohol-induced metabolic dysfunction, contribution to liver pathogenesis, the effect of pregnancy, and targeted therapy of ALD.

## Highlights

• Chronic intake of alcohol initiates a pathogenic process that involves the production of protein-aldehyde adducts, and release of cytokines.
• Involved gene polymorphisms may include alcohol dehydrogenase, cytochrome P4502E1, and those associated with alcoholism.
• Alcohol ingestion could be correlated to the risk of preterm births, there is no exact dose-response relationship.
• Oral drugs of pentoxifylline have reduced the severity of steatohepatitis in alcohol-use patients.

## 1 Introduction

Chronic and excessive alcohol consumption is a global healthcare problem, which leads to clinical illness and pathological changes causing alcohol-associated liver disease (ALD). ALD is associated with liver inflammation and injury or progressive fibrosis producing three major classes, each of which rarely exists in a pure form [1,2]. These include fatty liver, alcoholic hepatitis, and cirrhosis. Fatty liver is a feature that is present in most chronic drunkers and associated with chronic alcohol intake [3]. A significantly smaller percentage of alcohol users who drink heavily will progress on to alcoholic hepatitis, which is shown to be a precursor to cirrhosis of the liver [4,5]. The severe alcohol-associated liver disease has a very poor prognosis. The mortality of patients with alcohol-associated hepatitis who have related cirrhosis is more than 65 % at 5 years [[6], [7], [8]]. Even though alcohol is considered to be a direct hepatotoxin, it is just around 20 % of all patients with alcohol use disorder, who subsequently develop into alcohol-associated hepatitis [[9], [10], [11]]. Globally, around 150 million people are infected with hepatitis C virus (HCV) and every year increase the prevalence of death rate [12]. The reason for this is due to other comorbid factors that include immune status, heredity, age, nutritional factors, and gender [[13], [14], [15], [16]]. Both the duration and the quantity of alcohol intake are more important risk factors that are involved in the development of ALD. The pattern of the type of beverage and the pattern of drinking play a lesser role in determining the risk [[17], [18], [19]]. It appears that additional risk factors that are not yet well defined are required in the progress of hepatic injury beyond the fatty liver stage [[20], [21], [22]].

Corticosteroids are used for the treatment of ALD and recently due to the findings of linkage of tumor necrosis factor-alpha with ALD, more focus is given on antitumor necrosis factor antibodies. The previous study suggests that exposure to acetaldehyde from the alcoholic beverage, the acetaldehyde tends to offers the toxic effects of hypotension, tachycardia, facial flushing, and vomiting. Moreover, the higher percentage of alcohol consumption exhibited the effects of neurotoxicity and liver damage [23]. Antioxidants are also seen to produce a beneficial effect in ALD. However, there exists no proper treatment strategy that could treat any patients with ALD. This creates a gap in research. Our present review addresses the gap in research and summarizes the different drug therapy of ALD giving an outline of pathogenesis and diagnosis.

## 2 Alcohol consumption and ALD

While estimating alcohol consumption, it is to be understood that 12 fluid ounces of beer, four ounces of wine, or 1.5 fluid ounces of distilled spirits all contain approximately 14 g or 0.6 fluid ounces of pure alcohol [24]. Based on this, the threshold for developing severe alcohol-associated liver disease in men is an intake of less than 60–80 g per day of alcohol for 10 years [[25], [26], [27]], while women seem to be at an increased risk for developing the same degree of liver injury by consuming 20–40 g per day of alcohol [[28], [29], [30]].

As compared to men, women are more susceptible to ALD pathogenesis. Women tend to develop the advanced liver disease with less alcohol intake substantially [[31], [32], [33]]. The gender-dependent differences in the gastrointestinal and hepatic metabolism of alcohol are likely to contribute towards the increased susceptibility of women to alcohol-induced liver injury [34]. Furthermore, overexpression of TNF-α mRNA in the liver activates the liver cell functions and macrophages and this caused the pro-inflammatory response and ROS elevation to reveal the liver toxicity. The mechanism of liver toxicity has been reported with activation of ROS, IL-6, and IL-8 [35].

In addition to this are the poorly understood hormonal factors, immunological, social, nutritional, and host factors, all of which have been postulated to play a part in the development of the pathological process [36,37]. The known case of HCV prone to progressive liver disease such as liver cancer/liver cirrhosis [12]. Chronic Hepatitis C (HCV) infection is one of the important comorbidity factors in the progression of ALD to cirrhosis in chronic and excessive drinkers [[38], [39], [40]]. Alcohol intake of more than 50 g per day, significantly increases the risk of cirrhosis in individuals infected with HCV [[41], [42], [43]]. Patients with the alcohol-use disorder and HCV infection later go on to decompensated liver disease at a younger age, and this has a poorer overall survival [44,45].

As a sequela of the injurious processes overlapping secondary to alcohol abuse and HCV infection, the patients may develop an increased liver iron burden and, rarely, porphyria cutanea tarda [46,47]. In addition to these sequelae, alcohol intake in HCV-infected patients with cirrhosis increases the risk for the development of carcinoma of the liver [48]. A summary of risk factors causing ALD are given in Table 1.

**Table 1:** Risk factors for ALD.

**Quantity** • In men, 40–80 g per day of ethanol produces fatty liver; • 160 g per day intake for 10–20 years causes hepatitis or cirrhosis. • Only 15 % of alcohol users end up developing ALD. **Gender** • Women show greater susceptibility to ALD at quantity more than 20 g per day; two drinks per day are probably safe. **Viral infection** • Hepatitis C (HCV) and hepatitis B (HBV) infection that is concurrent with ALD is associated with rapid progression to fibrosis, cirrhosis, and even hepatocellular carcinoma • Causes of liver disease in the setting of HIV infection include viral hepatitis which interacts in an environment modulated by persistent immune activation and altered cytokine display. **Genetics** • Gene polymorphisms may include alcohol dehydrogenase, cytochrome P4502E1, and those associated with alcoholism. • Association of ALD and genetic linkage to explored a wide range of risks in the development of the liver disease. The linkage between ALD and rs738409 gene expression causes severe damage to liver functions including the exacerbation of liver cirrhosis. Lipid storage of liver via the activation of lipogenic transacetylase influenced/intermediate by the PNPLA3. The lipid accumulation of the liver strongly associates with the elevation of aminotransferase and resulting in inflammation of liver cells. Interaction between gene functions and ALD via GWAS to identify the mechanism of liver pathogenesis [58]. **Malnutrition** • Malnutrition is not a cause or requirement of alcohol injury. However, obesity and fatty liver from the effect of carbohydrate on the transcriptional control of lipid synthesis and transport may be factors. Attention should be given to nutritional support in patients.

## 3 Pathogenesis of ALD

**Fig. 1:**
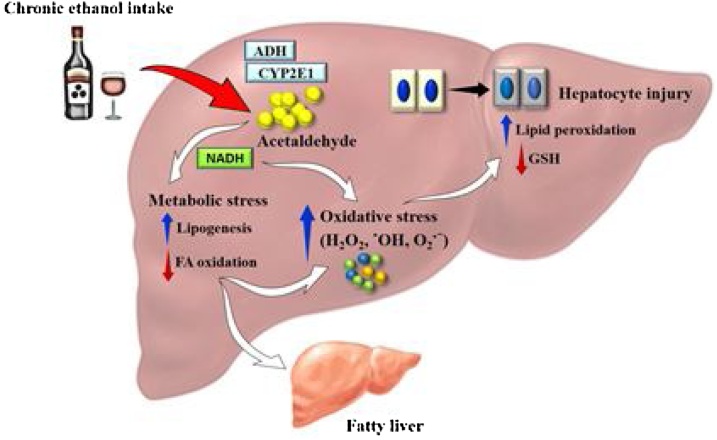
Hepatic metabolism of ethanol associated with oxidative stress.

Understanding of the detailed pathogenesis of ALD is quite incomplete to date [49,50]. Alcohol is a direct hepatotoxin, and its ingestion causes the initiation of numerous metabolic responses that influence the final hepatotoxic response [50,51]. The initial explanation of malnutrition as the major pathogenic mechanism has now given way to the present concept that the alcohol metabolised by the hepatocyte initiates a pathogenic process that involves production of protein-aldehyde adducts, immunologic activity, peroxidation of lipid, and release of cytokines [52]. Fig. 1 shows the hepatic metabolism of ethanol that contributes to enhanced oxidative stress in the body. In most cases, the time taken for liver disease to develop is directly related to the amount of alcohol consumed [[53], [54], [55]].

**Fig. 2:**
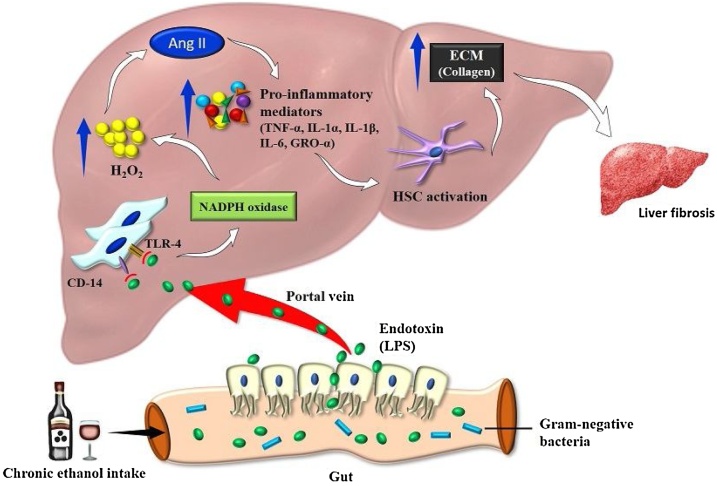
Pathogenesis of alcoholic liver disease.

Chronic consumption of alcohol produces a wide spectrum of hepatic lesions. Fatty liver (i.e., steatosis) is the earliest, most common response that develops in more than 90 percent of drinkers who consume 4–5 standard drinks per day. With continued drinking, alcohol-induced liver disease can proceed to liver inflammation (i.e., steatohepatitis), fibrosis, cirrhosis, and even liver cancer (i.e., hepatocellular carcinoma). The complex interaction of various distinct hepatic cell types is crucial to understand alcohol-mediated liver injury [56,57]. The main events in liver fibrogenesis include activation of stellate cells and production of collagen. The fibrosis that results due to this, determines the extent of damage to the architecture of the liver following chronic alcohol ingestion (Fig. 2).

The liver and gut microbiota axis linked into the bidirectional actions, and this involved the integration factors of genetic, dietary, and environmental. The alteration of the intestinal barrier explains the interrelations of the gut, liver and sometimes leads to negative effects on the liver. The intestinal microbes in hepatic disease are mainly associated with alcohol. This mutual reciprocal action established by the gut derivatives directly enters the liver and leads to intestinal secretions. Alcohol-induced microbial peptides increase the level of proinflammatory mediators in the liver surroundings and this interconnected with the epithelial barrier, the lining of the mucus membrane, and the gut microbiome. In the development of liver pathogenesis, alcohol directly acts on the liver parenchymal cells to initiate the abnormalities of intestinal barrier functions, alteration of microbiota, and increased toll-like receptors (TLRs) activation in liver cells. Especially, the alteration of gut microbiota to contribute to the pathogenesis of liver diseases [59,60].

### 3.1 Pathology

The response of the liver to any form of injury is quite limited. The initial and most common histologic response to hepatotoxic stimuli is fatty liver, and that includes excessive ingestion of alcohol. The fat that is accumulated within the perivenular hepatocytes actually coincides with the presence of alcohol dehydrogenase, which is the major enzyme that is responsible for alcohol metabolism. Continuous ingestion of alcohol leads to fat accumulation all through the entire hepatic lobule [61].

Although the extensive fatty change results in the hepatocytes being distorted with the accumulation of macrovesicular fat, stopping drinking results in the normalization of hepatic architecture and fat material inside the liver [62]. While ALD has been regarded as an entirely benign process, but similar to the features of nonalcoholic steatohepatitis, there are certain pathologic features such as perivenular fibrosis, giant mitochondria, and macrovesicular fat that may be associated with progression to further chronic liver injury [63,64]. The progress of liver injury from the stage of the fatty liver until the development of alcoholic-associated hepatitis is vague. The main hallmark of alcoholic hepatitis is an injury to hepatocytes that characterized by spotty areas of necrosis, ballooning degeneration, infiltrate with polymorphonuclear cells, and zones of fibrosis either bridging or in the perivenular and the perisinusoidal space or space of Disse [65]. Florid cases can show the presence of Mallory bodies, but these are neither specific nor it is necessary to establish the diagnosis. A precursor to the development of cirrhosis is alcoholic hepatitis. However, fatty liver and alcoholic hepatitis are potentially reversible once there is the cessation of drinking [66]. In half of the liver cirrhotic population, alcoholic hepatitis is seen and once cirrhosis occurs it is difficult to reverse even with abstinence from alcohol. However, abstinence can prevent further deterioration of the disease [67].

### 3.2 Clinical features

ALD has varied clinical manifestations. The clinical manifestation of alcohol-induced fatty liver is very subtle in its presentation and is characteristically detected as a consequence of the patient’s visit for a routine screening or employment screening or any unrelated issues. Previously unsuspected hepatomegaly is often the only clinical presentation. Occasionally, a patient with fatty liver is presented with right upper quadrant discomfort, tender hepatomegaly, nausea, and jaundice. Differentiation of alcohol-induced fatty liver and non-alcohol-induced fatty liver is difficult unless an accurate history of drinking habits, pattern, and quantity is obtained [68].

In every possible incidence where liver disease is present, a fully detailed drinking history should be elicited. Alcoholic hepatitis is usually associated with a wide range of clinical features. Cytokine production is thought to be the main factor responsible for all the systemic manifestations of alcoholic hepatitis. Fever, spider nevi, jaundice, and abdominal pain simulating an acute abdomen are the features that are seen at the end of the spectrum, while many patients will be entirely asymptomatic. Identification of the clinical features of alcoholic hepatitis is very important to initiate an effective, appropriate diagnostic and therapeutic plan [69,70]. Cirrhosis is a later stage of the ALD and the patient may experience weakness, fatigue, and weight loss. In the later stage, the patient may develop symptoms like jaundice, gastrointestinal bleeding, abdominal swelling, and confusion [71].

### 3.3 Laboratory features

The current diagnosis of ALD is based on the history of alcohol consumption, physical examination, and laboratory findings associated with alanine aminotransferase (ALT), aspartate aminotransferase (AST), and gamma-glutamyl transpeptidase (GGTP). The typical abnormalities seen in the laboratory results are nonspecific and include mild to moderate elevations of ALT, AST, and GGTP. This is often accompanied by hypercholesterolemia, hypertriglyceridemia, and sometimes hyperbilirubinemia. In alcoholic hepatitis and contrast to other causes of fatty liver, the AST and ALT are usually elevated fold [72]. Hyperbilirubinemia is quite common and is usually accompanied by mild to moderate increases in the levels of serum alkaline phosphatase. Defect in the function of hepatocyte synthesis indicates that the disease is more serious [73]. Coagulopathy and hypoalbuminemia are common in advanced stages of liver injury. An increase in the circulating neutrophil number to more than 5500/microL makes it similar to the event of the neutrophils infiltration observed in the extensive lesion of alcoholic hepatitis. Fatty infiltration of the liver and determining liver size are done best-using ultrasonography. Portal vein flow reversal, ascites, and intra-abdominal collaterals seen by ultrasonography indicate a serious liver injury and suggest little possibilities for the complete reversal of liver disease [74].

However, all these diagnostic parameters do not predict the degree of liver inflammation. Recent studies have developed several novel clinical criteria scores and biomarkers that provide better diagnostic, prognostic, and possible therapeutic options for the treatment of ALD. The evaluation of hepatoxicity is measured by different parameters such as serum aminotransferases, inflammatory mediators such as cytokines, DNA fragmentation, and investigation of histopathology [75]. Discriminant Function Index (DF); Glasgow Alcoholic Hepatitis Score (GAHS); the Age, Bilirubin, INR, and Creatinine (ABIC) score; and the Model of End-Stage Liver Disease (MELD) are some scores that are developed, validated, and used in clinical practice [76]. Recently, new biomarkers are developed that can non-invasively estimate the degree of alcohol intake and alcohol-induced liver damage. A summary of the recently investigated biomarkers is given in Table 2.

**Table 2:** Summary of recent biomarkers of ALD [76].

Biomarkers	Summary	Methods of analysing	Uses
Biomarkers of Liver Cell Death and Regeneration			
Cytokeratin 18	Intracellular intermediate filament Protein released during hepatocyte damage	ELISA for M65 and M30 (circulating fragments of cytokeratin-18)	Predicts diagnosis, severity and prognosis of alcoholic hepatitis
Augmenter of Liver Regeneration (ALR)	Protein that promotes liver regeneration, decreased in advanced liver disease	ELISA for serum levels of ALR	Human studies pending; could be used to predict staging of the severity in ALD

Biomarkers of Immune Response			
CD 163	Macrophage receptor protein on Kupffer cells, which are increased in AH	ELISA for plasma concentrations of soluble CD163	Potential to predict severity and prognosis of alcoholic hepatitis
ST2 Receptor	Protein receptors in inflammatory cascade found in hepatocyte inflammation and fibrosis	ELISA for plasma soluble ST2 receptors	Possible therapeutic target; predicts ongoing liver inflammation, staging of ALD severity
TNF- related Apoptosis inducing ligand (TRAIL)	Inflammatory cytokine released by the Kupffer cells activation, seen in hepatocyte injury	Western blot for serum levels of TRAIL	Potential to predict severity of alcoholic hepatitis; ongoing research a possible therapeutic target
Immunoglobulins (IgM, IgG, IgA)	Increased in AH	Quantitative serum immunoglobulin tests	Can be used to predict severity of alcoholic hepatitis;
MicroRNAs (MiR-155, MiR-223)	Noncoding RNAs that regulate expression of their respective target messenger RNA; miR-155 deficiency attenuates chronic alcohol-induced liver injury; MiR-223 is found in neutrophils and increased in AUD	Quantitative PCR for miRNA levels	Can be used to predict severity and prognosis of alcoholic hepatitis; ongoing research as possible therapeutic target

Biomarkers of Metabolic Changes			
Stearoyl-CoA desaturase 1 (SCD1)	Rate-limiting enzyme that catalyzes the formation of monounsaturated fatty acids and reduced lipid synthesis, influences hepatic inflammation	SCD1 activity can be measured indirectly by the palmitoleic acid to palmitic acid ratio via serum lipid measurements	Ongoing research as possible therapeutic target for early ALD
Magnesium	Electrolyte, which is decreased in alcohol use and liver disease	Serum levels	Could predict onset and staging of ALD
Uric acid	Breakdown product of purine metabolism, which is elevated in alcohol use and liver disease	Serum levels	Pro-inflammatory pathological could be used to predict severity in ALD

Biomarkers of Chemical Causes			
Acrolein	Toxic metabolite of alcohol metabolism, which accumulates in ALD	Urine tandem mass spectrometry detects the catabolic product of acrolein, 3 hydroxypropyl mercapturic acid (3HMP)	Can be used to predict severity of alcoholic hepatitis
Resolvins	Lipid mediators that counter-regulate proinflammatory responses, decreased in ALD	ELISA for serum levels of resolvins	Could predict inflammation and severity of alcoholic hepatitis; ongoing research as possible therapeutic target

## 4 Preterm and alcohol

Preterm birth is diagnosed if delivery occurs between 20 and 37 weeks of gestational age. In a minority of cases, this is iatrogenic as a result of doctors inducing delivery before the expected date due to maternal conditions such as pre-eclampsia and gestational diabetes or fetal conditions such as fetal growth restriction where the benefits of the baby being delivered outweigh that of the remaining in the uterus. However, the majority of preterm births are due to spontaneous labor or is the complication of preterm premature rupture of membranes [77].

Among the many factors considered for preterm birth, substance abuse or alcohol ingestion is considered to be the main [78]. The National Addiction survey in Mexico showed that women classified as suffering from Alcohol Dependence Syndrome had a very high risk of low birth weight and/or preterm delivery [79]. Another large study in Japan known as the Japan Environment and Children's Study (JECS) which looked at over 94000 singleton pregnancies showed that alcohol consumption during the second and third trimesters was associated with an increased risk of preterm delivery. The risk was also increased, according to the level of alcohol ingestion [80,81].

**Fig. 3:**
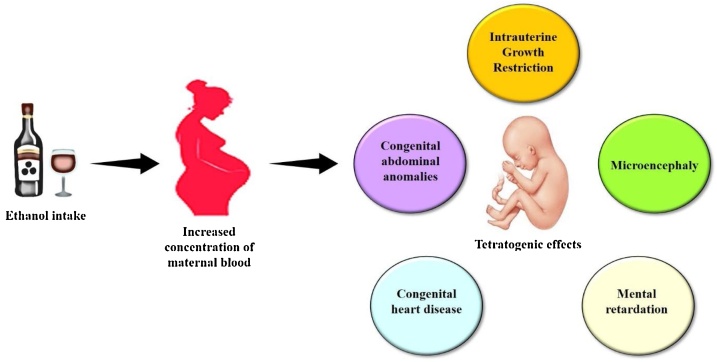
Effect of alcohol in pregnancy.

While the level of alcohol ingestion could be correlated to the risk of preterm births, there is no exact dose-response relationship between the amount of alcohol consumed during the prenatal period and the extent of damage caused by alcohol in the infant. The alcohol crosses the placenta without restriction [82]. The level of alcohol in the fetal blood can equate to the level in the mother after two hours. The maternal metabolic capacity to eliminate alcohol varies substantially between women, which may explain the varying effects of alcohol in women. Routine screening for urine alcohol levels for all antenatal patients is not cost-effective. Alcohol is associated with a higher risk of preterm births and other disabilities (Fig. 3). However, patients with these risk factors often have other risks for preterm birth, such as poor socioeconomic and nutritional status and anemia which may also have contributed to preterm births [83].

Consumption of alcohol during pregnancy causes movement of alcohol through the umbilical cord which then passes to the fetus. Increase concentration of maternal blood alcohol leads to teratogenic effects, including miscarriage, intellectual disabilities, microencephaly, and congenital heart disease. These defects are also known as fetal alcohol spectrum disorders (FASD) [84,85].

National guidelines from many countries recommend complete abstinence from alcohol during pregnancy to avoid both maternal and fetal adverse effects. Alcohol is a teratogen that impacts fetal growth and development at all stages of pregnancy [85]. Identification and counseling of women who use alcohol can decrease intake during pregnancy. Several screening tools are available to screen pregnant women. Clinicians should use one that is appropriate for their setting and population. Intervention sessions with counseling can be done for those who are not heavy drinkers. It should be remembered that any pharmaceutical interventions for the prevention or amelioration of adverse alcohol effects on the mother or fetus will not be useful [86].

## 5 Treatment approach of ALD

**Fig. 4:**
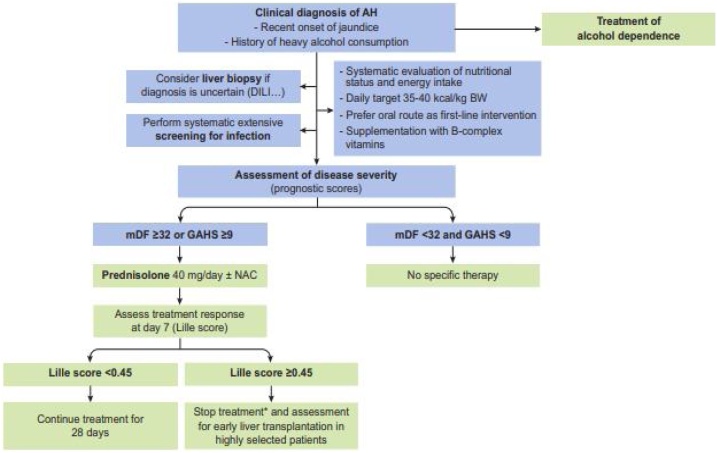
Treatment algorithm for the treatment of patients with alcoholic hepatitis. *Particularly in null responders (Lille score ≥0.56). AH, alcoholic hepatitis; BW, body weight; DILI, drug-induced liver injury; GAHS, Glasgow alcoholic hepatitis score; mDF, Maddrey discriminant function.

Many drugs are studied for their use in the pharmacotherapy of ALD, but none of the drugs has proven to be safe. Additionally, these drugs can enhance liver toxicity [87]. The current treatment algorithm for the treatment of alcoholic hepatitis as per the European Association for the study of the Liver (EASL) guidelines is given in Fig. 4 (EASL guidelines, 2019).

A combination of ribavirin and peginterferon exhibits rare autoimmune adverse effects in hepatitis C and the effect is severe. The cause of autoimmune adverse effects basically from the inappropriate intake of antiviral drugs and these conditions immediately to seek the specific treatment. Combination of these antiviral drugs possessing the autoimmune adverse events and this combination elevated the levels of IgM and IgG. Moreover, there is no linkage between autoimmune adverse events and interferon use [12].

**Fig. 5:**
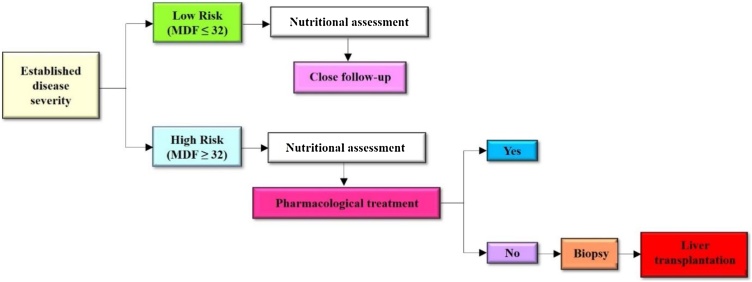
Treatment strategies for alcohol-associated liver disease.

Till now baclofen has been proven safe for the treatment of patients with alcohol-use disease but other drugs suggested by the Food and drug administration (FDA) such as disulfiram and naltrexone can induce hepatotoxicity. So those are not a well-appropriate treatment option for alcohol-induced liver disease patients. Opioids and nalmefene are also FDA approved for the treatment of liver disease, but the safety of these drugs excluded from the registration trial [88]. The treatment strategies for ALD are shown in Fig. 5.

### 5.1 Nutritional supplements

The treatment of liver disease is limited to alcohol-induced liver disease. The treatment of liver disease is more integrated among health care providers, including physicians and psychologists to achieve abstinence, or at least to reduce the risk of liver toxicity [89]. For those patients with alcohol-induced liver diseases, several clinical studies reveal that one of the major causes is malnutrition, which exacerbates the severity of the liver disease.

Many chronic alcoholics are malnourished, it may be either due to intake of too few essential nutrients (like carbohydrates, proteins, fats, and vitamins) or due to the metabolism of alcohol which prevents the body from digesting and utilizing essential nutrients. Also, previous studies reported roughly 60 % of cirrhosis patients had a severe condition of malnutrition [90,91].

According to the severity of the liver disease, the patient risks are categorized into different levels. Initial support with nutritional supplements includes zinc, magnesium, vitamin D, E, and B1 (listed in Table 3) which is followed by pharmacological treatment, which includes mycophenolate mofetil (the drug that reduces liver inflammation) and other drugs listed in Table 4. In the severe condition of liver cirrhosis, it is suggested to go for orthotopic liver transplantation (OLT) with appropriate precautionary measures.

**Table 3:** Supplementation of nutritional substances.

Clinical syndrome	Recommended clinical indication
Inflammatory disease, diarrhoea and immunodeficiency	Zinc [95]
Muscle spasm and glucose intolerance	Magnesium [96]
Osteopenia and osteomalacia	Vitamin D [97]
Antioxidative	Vitamin E [98]
Anemia and cancer risk	Folate [99]
Nuropsychiatric symptoms	Niacin [100]
Cardiomyopathy and Wernicke-Korsakoff encephalopathy	Thiamine [101] Vitamin B1 Thiamine Deficiency (Beriberi).
Myopathy and cardiomyopathy	Selenium [102]

**Table 4:** Current targeted treatment for ALD.

Drug name	Drug action	Uses
Mycophenolate mofetil	Immunosuppressor and IL-1 blocker	Inhibition of hepatic inflammation [107,108]
Emricasan	Pan-caspase inhibitor	Inhibition of apoptosis [109]
Anakinra	Blocking of IL-1 receptor	Inhibition of inflammation [110]
Lactobacillus rhamnosus	Probiotic	Inhibition of bacterial overgrowth and reduce the inflammatory response [111]
Obeticholic acid	Biliary acid	Improved cholestasis [112]
Stem cells	Progenitors	Promote liver regeneration [113]
Rifaximin	Used as antibiotic	Inhibition of overgrowth of bacteria and prevent the infection [114]
Metadoxin	Antioxidant	Protection of liver [115]
Amoxicillin clavulanate	Antibiotic	Inhibition of overgrowth of bacteria and prevent the infection [116]
Garlic acid	Antioxidant	Protection of liver [117]
Zinc	Oligoelement	Reduce the gut inflammation [118]
Ciprofloxacin	Antibiotic	Inhibition of overgrowth of bacteria and prevent the infection [119,120]

Additionally, the supplementation of nutritional substances helps to protect liver toxicity. Nutritional supplements also provide protein balance and protected from alcohol-induced encephalopathy which is maintained by amino acids such asvaline, leucine, and Isoleucine. These are supportive/preventive treatments of alcohol-induced liver disease [92].

Moreover, the current guidelines suggest the intake of protein and calories of 1.2–1.5 g and 30–35 kcal/g respectively. Supporting these values most of the ALD patients have been diagnosed lower range than the normal values. Therefore, nutritional supplementation is needed for ALD patients to reduce the risk of liver cirrhosis [93]. Consideration of micronutrient deficiencies in alcohol-induced liver disease (Table 2), the following are affected by chronic alcohol consumption and advised to maintain these essential substances in ALD [94].

The prevalence of malnutrition reaches almost 100 % in severe alcoholic hepatitis patients. The earlier studies reflected the possible benefits of nutritional support with hepatitis and cirrhosis patients [103,104]. Subsequently, less calorie intake leads to lethal effects that emphasize the patient's need for proper nutritional balance.

### 5.2 Drugs

So far there is no established drug available in the market that could cure ALD; however, for the management of ALD many drugs are available (Table 4) [105]. Healthcare providers strongly believe that abstinence from alcohol is the best choice to prevent ALD. Several reports demonstrated that an oral drug of pentoxifylline has reduced the severity of steatohepatitis in alcohol-associated disease patients. Recent studies stated that N-acetylcysteine (NAC) with corticosteroids provides better recovery effects in alcohol-induced liver disease [106].

### 5.3 Current clinical trials on drug development for ALS

It is time to look freshly into the drug targets for the treatment of ALS. Over the last few years, National Institute on Alcohol Abuse and Alcoholism (NIAAA) has encouraged to find new targets conducting different clinical trials. Based on the trials, the new drugs are categorized into four types: a) Drugs that act on the gut axis, b) antiinflammatory agents, c) antioxidants, and d) Drugs with regenerative benefits. The current clinical trials with novel therapeutic agents for the treatment of ALS, particularly alcoholic hepatitis is shown in Table 5 [121].

**Table 5:** Current clinical trials with novel therapeutic agents for treatment of alcoholic hepatitis.

Pharmaceutical agent	Mechanism of action	Study design	Main inclusion	Primary endpoint	Status
Bovine colostrum (IMM124E)	IgG to LPS and reduces bacterial translocation	Placebo controlled RCT	MELD score ≥20 but ≤28	Decrease in serum endotoxin levels at 7 months	Phase II, active, not recruiting
Lactobacillus rhamnosus GG	Change in gut microbiome	Placebo controlled RCT	MELD score <21	Change in MELD score at 30 days	Phase II, active and recruiting
Augmentin	Antibiotic amoxicillin plus clavulanic acid	Placebo controlled RCT with CS	MELD score ≥21	Survival at 2 months	Phase II, active and recruiting
Faecal transplant	Change in gut microbiome	RCT FMT vs. CS	Eligible for CS treatment	Survival at 3 months	Phase II, active and recruiting
Anakinra	Antagonist to IL-1 receptor	RCT Anakinra + Zn + PTX vs. CS	MELD score ≥20 and Madre DF ≥32	Survival at 6 months	Phase II, active and recruiting
Obeticholic acid [INT-747]	FXR activation, bile acid agonist, and anti-inflammatory	Placebo controlled RCT	MELD score >11 and <20	Change in MELD score at 6 weeks	Phase II, completed
Selonsertib [GS-4997]	ASK-1 antagonist to inhibit MAPK, JNK, p38	Placebo controlled RCT with CS	Maddrey DF score ≥32	Safety and SAE at 28 days plus 30 days	Phase II, completed
Emricasan [IDN-6556]	Pan caspase inhibitor	Placebo controlled RCT	MELD score >20 but <10	Survival at 28 days	Phase II, terminated after 5 patient
Metadoxine	Antioxidant and promotes abstinence	Placebo controlled RCT with CS	Severe alcoholic hepatitis	Survival at 30 days	Phase IV, completed
IL-22 [F-652]	Anti-inflammatory and hepatic regeneration	Open label	MELD score 11–28	Safety and SAE at 42 days	Phase I completed Phase II planned
G-CSF [Filgrastim]	Increase neutrophils, hepatic regeneration	Placebo controlled RCT with CS in partial responder and without CS in null responder	Maddrey DF score ≥32	Survival at 2 months in null responder to CS and at 6 months in partial responder	Phase IV, active and recruiting

CS, corticosteroid; MELD, model for end-stage liver disease; PTX, pentoxifylline; RCT, randomised controlled trial; SAE, serious adverse event; SOFA, sequential organ failure Assessment [78].

### 5.4 Liver transplantation

Orthotopic liver transplantation (OLT) is one of the popular treatments in severe ALD [122]. After OLT, the survival of the patients linked with cardiovascular disease and de nova malignancies. This linkage strongly suggests a habit of cigarette smoking among the transplanted patients [123]. OLT patients need to monitor the linkage of cigarette smoking-induced mortality in pre and post-surgery. Previous studies suggest that the OT is an acceptable treatment for ALD patients, those who are severely affected by the normal liver function. Moreover, after the OLT the patients must be given the proper diet habits and need lifelong follow-up to avoid complications [124]. Due to chronic alcohol abuse, the patients can cause multisystemic effects and associated diseases such as malnutrition, vitamin deficiencies, muscle wasting, neurological abnormalities, myopathy, etc. Ideally, based on the patient experience and clinical studies, chronic alcohol abuse patients require multidisciplinary approach treatment [125,3].

## 6 Conclusion

Based on the epidemiological and clinical diagnosis of patients with the alcohol-use disorder, the complete treatment is still disappointing. The deficiency of treatment may be influenced by the lack of pharmaceutical approaches in the treatment of alcohol-induced liver disease and required to initiate the awareness program by the governments and health care providers. Moreover, alcohol abuse fully is preventable, so we need to educate the importance of alcohol abstinence among alcohol abuse patients.

## Author contributions

VS, SC and RJ was responsible for the conception and design of the review. VS, SC, RJ, NKF, SF, IH, AD and WYS collected literatures. VS, NKF, SF, IH, WYS and AD analyzed literatures. VS, SC and RJ drafted the manuscript and WYS, VS and IH drew the figures.

## Declaration of Competing Interest

The authors declare no conflict of interest.
